# In Silico Study: Combination of α-Mangostin and Chitosan Conjugated with Trastuzumab against Human Epidermal Growth Factor Receptor 2

**DOI:** 10.3390/polym14132747

**Published:** 2022-07-05

**Authors:** Sandra Megantara, Nasrul Wathoni, Ahmed Fouad Abdelwahab Mohammed, Cecep Suhandi, Maryam H. Ishmatullah, Melisa F. F. D. Putri

**Affiliations:** 1 Department of Pharmaceutical Analysis and Medicinal Chemistry, Universitas Padjadjaran, Sumedang 45363, West Java, Indonesia; cecep17001@mail.unpad.ac.id (C.S.); maryam17002@mail.unpad.ac.id (M.H.I.); melisa17001@mail.unpad.ac.id (M.F.F.D.P.); 2 Department of Pharmaceutics and Pharmaceutical Technology, Universitas Padjadjaran, Sumedang 45363, West Java, Indonesia; nasrul@unpad.ac.id; 3 Department of Pharmaceutics, Faculty of Pharmacy, Minia University, Minia 61519, Egypt; ahmed.mohamed1@minia.edu.eg

**Keywords:** breast cancer, α-mangostin, trastuzumab, HER2, in silico, FireDock

## Abstract

Breast cancer is a type of cancer with the highest prevalence worldwide. Almost 10–30% of breast cancer cases are diagnosed as positive for HER2 (human epidermal growth factor receptor 2). The currently available treatment methods still exhibit many shortcomings such as a high incidence of side effects and treatment failure due to resistance. This in silico study aims to simulate α-mangostin and chitosan combination conjugated to trastuzumab formulation against HER2 as an effort to improve breast cancer patient therapy. This molecular docking simulation was done through using PatchDock Server. The materials used including the two-dimensional structure of α-mangostin, chitosan, and sodium tripolyphosphate from the PubChem database; trastuzumab FASTA sequence from the DrugBank database; and HER2 structure obtained from a crystal complex with PDB ID: 1N8Z. The results indicated that the particle of α-mangostin and chitosan combinations interacted mostly with the crystallizable fragment (Fc region) of trastuzumab in the conjugation process. The conjugation of trastuzumab to the particle of a combination of α-mangostin and chitosan resulted in the greatest increase in the binding score of the smallest-sized particles (50 Å) with an increase in the score of 3828 and also gave the most similar mode of interaction with trastuzumab. However, the conjugation of trastuzumab eliminated the similarity of the mode of interaction and increased the value of atomic contact energy. Thus, a cominbation of α-mangostin and chitosan conjugated to a trastuzumab formulation was predicted can increase the effectiveness of breast cancer therapy at a relatively small particle size but with the consequence of decreasing atomic contact energy.

## 1. Introduction

Breast cancer is a type of cancer with the highest prevalence in the world. The World Health Organization (WHO) estimates that 2.1 million women in the world suffer from breast cancer each year. In fact, in 2018 it was recorded that as many as 627,000 people in the world died from breast cancer (about 15% of total breast cancer cases) [1].

The treatment of breast cancer still relies on chemotherapy, radiation therapy, and surgery [2]. Currently available chemotherapy drugs are estrogen receptor inhibitors, monoclonal antibodies against human epidermal growth factor receptor 2 (HER2), tyrosine kinase inhibitors, and various other mechanisms [3,4,5]. However, these still have many shortcomings, such as a high incidence of side effects and the failure of therapy due to resistance [6,7].

Active compounds sourced from herbal plants can be an alternative in overcoming the lacks of drugs available in the treatment of breast cancer. α-mangostin, which is a xanthone derivative compound obtained from the pericarp of the mangosteen fruit (*Garcinia mangostana* L.), was known to be a promising anti breast cancer agent [8,9,10]. α-mangostin was known to have anticancer activity through the mechanism of the inhibition of the fatty acid synthase (FAS) and the induction of apoptosis in the cell signaling pathway (FAK, ERK1/2 and AKT) against MCF-7 and MDA-MB-231 cell lines [11]. However, α-mangostin has drawbacks in terms of solubility, as well as selectivity to target cancer cells [12,13,14].

Nowadays, polymeric nanoparticle-based drug delivery systems have become one of the answers to various drug formulation problems. Particle combination-based drug delivery can increase the solubility of an active pharmaceutical substance, especially phytochemical compounds [15,16,17,18]. One of its applications is the use of chitosan as a biodegradable polymer, widely used in increasing the efficiency and efficacy of active pharmaceutical substances delivery [10]. A study concluded that α-mangostin was successfully formulated into nano-polymeric form (nano-sized combined particles) using chitosan with particle size characteristics in the range of 200–400 nm [19].

On the other hand, the conjugation of drug delivery packages to specific target receptor compounds can increase the selectivity and effectiveness of breast cancer therapy [20]. Selective targeting of drugs to a specific targets can be implemented by conjugating drug delivery packages with target-specific ligands to be directed to an appropriate receptor, which are overexpressed on the surface of the cancer cells of interest [21]. Trastuzumab, as an anti-HER2 that is commercially available and that has been approved by the Food and Drug Administration (FDA), can be used as a suitable target ligand for the α-mangostin nano-polymeric system against cancer cells with HER2 expression [22,23]. The decoration of drug delivery packages with trastuzumab is known to increase the cellular uptake of conjugated packages mediated by the specific interactions of trastuzumab and HER2 through an endocytosis mechanism [23,24].

Thus, the formulation of the combination of α-mangostin and chitosan conjugated with trastuzumab can be an alternative answer to the problems that have been stated previously. In silico assay based on molecular docking was carried out as a preliminary test in predicting the molecular interactions that occur between the preparations made with HER2 as the target. In addition, the results of in silico testing can be used as a guide in formulation and testing in a wet laboratory (in vitro, in vivo, or ex vivo).

## 2. Materials and Methods

### 2.1. Materials

The hardware used was a personal computer (PC) with AMD^®^ A8 APU specifications and 4 gigabytes of RAM. The in silico assay was performed using software that can be accessed online or downloaded for free for academics. The software used includes:

1. Program ChemOffice 2012 (PerkinElmer Informatics, Waltham, MA, USA) contains the program ChemDraw 12.0 and Chem3D 12.0 was used to draw a 2D structure and 3D structure of the molecule [25];
2. The BIOVIA Discovery Studio 2017 (BIOVIA, San Diego, CA, USA) program was used to visualize the structure of molecules, proteins, or complexes resulting from molecular docking simulations and the extraction of molecular structures or compounds [26,27];
3. The PACKMOL-Memgen (IQ-UNICAMP, University of Campinas, Campinas, SP, Brazil) program was used to package α-mangostin, sodium tripolyphosphate, and chitosan compounds into combined particle form [28];
4. The SWISS-MODEL program (Swiss Institute of Bioinformatics Biozentrum, Klingelbergstrase, Basel, Switzerland), which was accessed online from 2nd–20th February 2021 via https://swissmodel.expasy.org/ (accessed on 20 April 2022), was used in the modeling and validation of trastuzumab structure [29,30];
5. The PatchDock program (Sackler Institute of Molecular Medicine, Tel Aviv University, Tel Aviv, Israel) was accessed online on 2nd–20th February 2021 at https://bioinfo3d.cs.tau.ac.il/PatchDock/ (accessed on 12 March 2022) was used for the trastuzumab conjugation process and molecular docking simulation [31,32];

The materials used include the three-dimensional structure of HER2 downloaded from the Protein Data Bank (PDB) (http://www.rscb.org/ (accessed on 3 February 2022)) with ID code 1N8Z, the 2D structure of α-mangostin, sodium tripolyphosphate, and chitosan obtained from the PubChem database (https://pubchem.ncbi.nlm.nih.gov/ (accessed on 15 February 2022)), and trastuzumab amino acid sequence (FASTA) obtained from the DrugBank database (https://go.drugbank.com/ (accessed on 20 April 2022)).

### 2.2. Methods

#### 2.2.1. Structure Preparation of α-Mangostin, Sodium Tripolyphosphate, and Chitosan

The two-dimensional structure of α-mangostin, sodium ion, tripolyphosphate ion, and chitosan was drawn using the ChemDraw 12.0. The structure of sodium tripolyphosphate was drawn separately in the form of sodium and tripolyphosphate ions, while the structure of chitosan was drawn in the form of ionized in the amine group (–NH_2_ becomes –NH_3_^+^). It is based on the α-mangostin nano-polymeric formulation process that converts the constituents of the particles into their ionized form [19]. The two-dimensional structure of each compound was then saved in a file in .cdx format. Then the optimization of the structure into a three-dimensional form through the energy minimization of molecular mechanic 2 (MM2 minimization) using Chem3D 12.0 program and saved in a file with the .pdb format.

#### 2.2.2. Packaging of α-Mangostin, Sodium Tripolyphosphate, and Chitosan into Combined Particles

The particle shape of the combination of α-mangostin and chitosan was made using the PACKMOL-Memgen program by inputting the number of molecules from each constituent of the combined particle. The combination of α-mangostin and chitosan particles was made in three sizes with radii of 50 Å, 75 Å, and 100 Å. The composition of each constituent was based on their respective weights used in the formulation process that has been carried out previously; they are α-mangostin 20 mg (410.5 g/mol), chitosan 200 mg (1526.5 g/mol), and sodium tripolyphosphate 28 mg (367.86 g/mol) [19]. The amount of each constituent molecules obtained through the conversion of mass into Avogadro’s number of molecules by an equation that describes the relationship between the weight (m), relative molecular mass (Mr), and the Avogadro constant (N_A_), as follows [33]:

Number of Molecules = (m/Mr) × N_A_ N_A_ = 6.02 × 10^23^ (1)

Through this conversion, the particle composition of the combined particles was used as attached in Table 1 to be processed using the PACKMOL-Memgen program, and then the results are saved in a file in .pdb format.

#### 2.2.3. Structure Modeling and Validation of Trastuzumab

The three-dimensional structure of trastuzumab was prepared using the SWISS-MODEL Server by inserting the previously obtained FASTA sequences. The three-dimensional structure modeling of trastuzumab was carried out using adjustments based on the system’s automatic search for suitable templates. The modeling results were then validated by analyzing the GMQE (global model quality estimation) value, which offers the degree of similarity between the structures made to the template and QMEAN |Z-score|, which indicates the degree of the naturalness of the structure obtained. GMQE value close to 1 and QMEAN |Z-score| close to 0 indicate the good structural quality of the modeling results [30,34].

#### 2.2.4. HER2 Preparation as Receptor

The HER2 structure as a target receptor was obtained by extracting the HER2 structure from the HER2 complex (extracellular domain) and trastuzumab (Fab region) at PDB ID: 1N8Z using the BIOVIA^®^ Discovery Studio 2017 program (BIOVIA, San Diego, CA, USA). The three-dimensional structure of HER2 was then saved in a file in .pdb format.

#### 2.2.5. Particle Conjugation of α-Mangostin and Chitosan Combination against Trastuzumab

Particles of the previously prepared combination of α-mangostin and chitosan were conjugated with trastuzumab via molecular docking simulation using PatchDock Server. The simulation was completed by entering a file with the .pdb format of each structure into the server. The combined particle of α-mangostin and chitosan was then regulated as a ligand, whereas trastuzumab was regulated as a receptor. Then, the best conformation was selected from the results of the molecular docking simulation based on the docking score obtained. The conjugated structure of each particle of the combined particle was then saved in a file in .pdb format.

#### 2.2.6. Molecular Docking Simulation of Particles of a Combination of α-Mangostin and Chitosan Conjugated with Trastuzumab against HER2

This stage is carried out using PatchDock Server by entering a file in .pdb format with HER2 as a receptor and test structure and standard as a ligand. The test structure was a combined particle structure of α-mangostin and chitosan conjugated with trastuzumab with certain size variations (50 Å, 75 Å, and 100 Å). While the standards consist of constituents that are not processed or broken down during the delivery of the molecular package, including α-mangostin, tripolyphosphate ion, ionized chitosan, trastuzumab, and combined particles of α-mangostin and chitosan with variations in size (50 Å, 75 Å, and 100 Å, respectively). The results of the molecular docking simulation include docking score, area, and ACE (atomic contact energy) [35,36].

## 3. Results

The preparation of the molecular structure of α-mangostin, chitosan, and sodium tripolyphosphate based on the PubChem database using ChemDraw 12.0 and Chem 3D 12.0 programs was successfully carried out, as described in Table 2. The three-dimensional structure of each compound was obtained through an energy-minimization process. This energy-minimization process was aimed to obtain a three-dimensional conformation of the compound structure as it occurs naturally [37,38].

**Figure 1:**
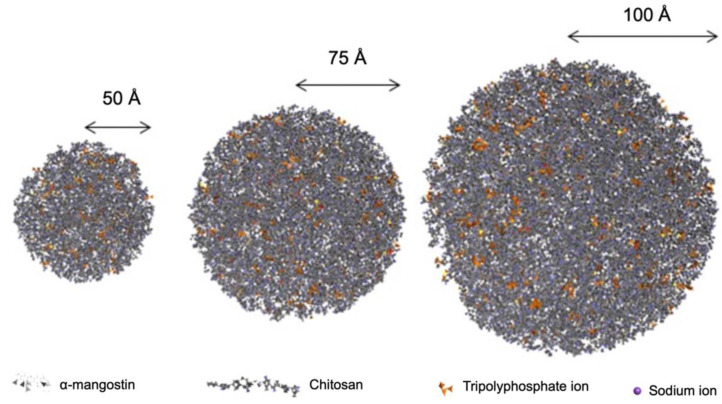
Combined particles of α-mangostin, sodium tripolyphosphate, and chitosan.

The structure of the prepared compound was then packaged into a combination of α-mangostin and chitosan particles using the PACKMOL-Memgen program. The PACKMOL-Memgen program is a program that is used to package a collection of molecules of known composition into the desired shape [28,39]. Based on the nano-polymeric characterization study of α-mangostin conducted by Wathoni et al., the combination formulation of α-mangostin and chitosan with the composition of α-mangostin 20 mg, chitosan 200 mg, and sodium tripolyphosphate 28 mg, resulted in a number of combined particles of α-mangostin and chitosan with size varying in spherical shape. The true size of the combined particles produced in the study of Wathoni et al. is in the range of nanoparticle-size (267.12 nm of diameter) [19]. However, PACKMOL-Memgen does not have the capacity to pack molecules down to nano size. Thus, in this study, a size variation approach was used to see the effect of size on combined particles on its interaction with HER2. The structure of the packaging results was described as combined particles with size variations of 50 Å, 75 Å, and 100 Å that can be seen in Figure 1.

**Figure 2:**
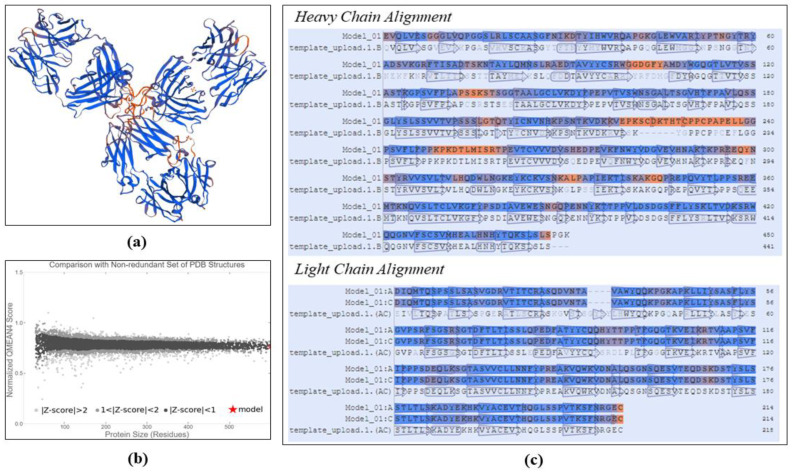
Trastuzumab modeling, resulting in: (**a**) 3D structure of trastuzumab, (**b**) comparison with non-redundant set of PDB structures, (**c**) heavy and light chain alignment.

As the three-dimensional structure of trastuzumab cannot be retrieved due to its unavailability in the protein databank, trastuzumab was modeled by using a SWISS-MODEL server to generate the three-dimensional structure of trastuzumab, as depicted in Figure 2a. The structure of trastuzumab was generated by arranging the FASTA sequence of trastuzumab from DrugBank with the structure that has the most similar structure. SWISS-MODEL gave an automatic searching result for trastuzumab’s template that showed that pembrolizumab (IgG4) had the highest similarity with the structure of trastuzumab, about 82.60%. In Figure 2c is depicted the alignment of the amino acid residues that made up trastuzumab and its template. The validity of the trastuzumab modeling results can be assessed based on the GMQE and QMEAN |Z-score| values as presented on Table 3. GMQE rated as 0.85 (close to 1) indicates that the structure obtained has a good conformation proximity to the template. The QMEAN |Z-score| value of 0.05 (close to zero) indicates that the modeling results in a conformation close to the natural structure of trastuzumab. The reliability of the modeled trastuzumab structure can also be evaluated based on the position of the QMEAN |Z-score| value on the distribution diagram as depicted in Figure 2b. The QMEAN |Z-score| value that was obtained from the modeling was located in a position wherein the protein structure has a high degree of reliability.

## 4. Discussion

**Figure 3:**
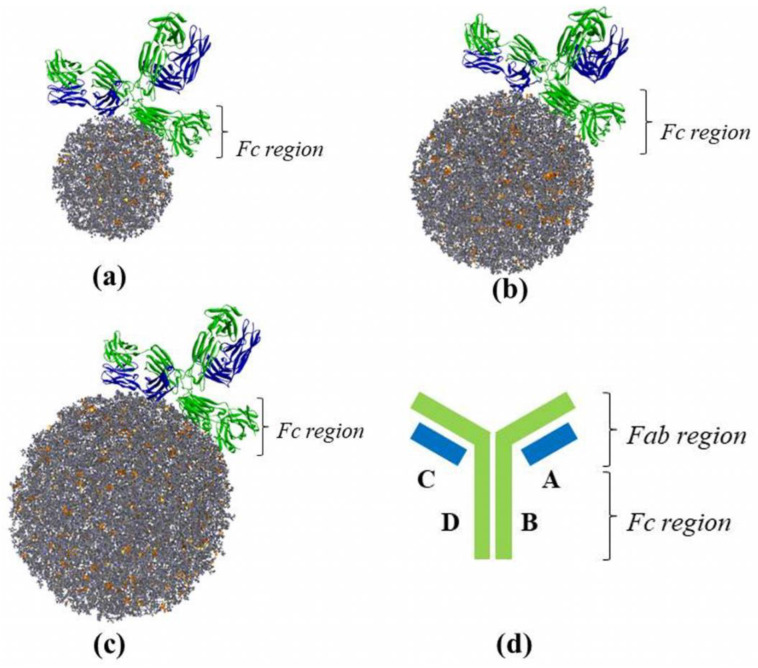
Combined particle-size variation conjugated to trastuzumab: (**a**) 50 Å, (**b**) 75 Å, (**c**) 100 Å, and (**d**) the regional structure of trastuzumab.

The particle structure of the combination of α-mangostin and chitosan that has been made was then conjugated with trastuzumab through a molecular docking simulation process using PatchDock Server. Figure 3 describes the structure of the conjugated results of each particle-size combination of α-mangostin and chitosan with trastuzumab. The conjugates obtained showed that the combined particle structure interacted with the crystallizable fragment site (Fc region) in the B and D chains of trastuzumab. These results are consistent with the results of research conducted by Yousefpour et al., which conjugates the nano-polymeric of doxorubicin with trastuzumab. In this study, the doxorubicin nanopolymeric structure made using chitosan polymer also interacted with the Fc region of trastuzumab in the formation of its conjugate [40]. However, the particles also exhibit hydrogen interactions with the C chain (Fab region). As attached in Table 4, the combined particles with a radius of 50 Å provide hydrogen interactions with the amino acid residues Gln147, Asn152, Thr197, Ser202, and Ser203.

While the combined particles with a radius of 75 Å showed hydrogen interactions with Asp151, His198, Leu201, Ser203, and Pro204 in the C chain of trastuzumab. Then, the combination particles with a radius of 100 Å offered hydrogen interactions with Ser12, Thr109, Val110, Tyr140, Lys149, Glu195, Gln199, Ser202, Ser203, and Thr206 in the C chain of trastuzumab. This phenomenon can occur due to the interaction of the combined particle molecules in the Fc region occurring on the side of the trastuzumab, which causes the Fab region of the trastuzumab to have an ideal atomic distance to form hydrogen interactions with the combined particle molecules.

The results of molecular docking simulation of the test and standard compounds were attached in Table 5. The combined particle (100 Å) conjugated with trastuzumab had the highest binding score of 29,996. However, the effect of trastuzumab conjugation on the combined particles was seen significantly in the combined particle size of 50 Å. For the combined particle size of 50 Å and 100 Å, the docking score increased by 3828 and 112. As for the combined particle size of 75 Å, the conjugation of trastuzumab resulted in a decrease in the bond score of 1338. These results indicate that the large effect of trastuzumab conjugation can be more pronounced at the smaller combined particle size.

Based on the molecular interactions that occur as shown in Table 6, the combined particle size of 50 Å that was not conjugated with trastuzumab had the most similar mode of interaction with the mode that occurs in trastuzumab. It shows similar hydrogen bond motives in common with that of trastuzumab compared to other test compounds. Based on the molecular interactions that occur, trastuzumab exhibits hydrogen interactions with amino acid residues Asn89, Glu216, Tyr252, Cys289, and Glu330 in HER2. Meanwhile, the combined particle with a size of 100 Å did not result in the same hydrogen interaction as happened in trastuzumab. Meanwhile, the combined particles size of 50 Å and 75 Å still showed some hydrogen bond interactions at the same amino acid residue, namely in the combined particle size of 50 Å towards Asn89, Tyr252, and Glu330 and on the combined particle size of 75 Å only toward Tyr252. As for each particle size, the combination conjugated with trastuzumab did not give the same hydrogen bond interaction with HER2 as trastuzumab did. These results indicate that the larger the particle size and the conjugation effects of trastuzumab shifts the active site of the combined particle to HER2, which is different from the interaction of trastuzumab and HER2. Thus, it can be concluded that obtaining optimal pharmacological effects (preparations interact with HER2 as happened on trastuzumab) can be achieved by reducing the size of the combined particle of α-mangostin and chitosan to be made.

## 5. Conclusions

The conjugation of the combined particle of α-mangostin and chitosan with trastuzumab was successfully carried out. The combined particles were known to interact with trastuzumab on the crystallizable fragment site (Fc region) of trastuzumab. However, there are also a number of hydrogen interactions between the combined particles and the fragment antigen-binding site (Fab region) of trastuzumab. Molecular docking simulations of the combined particles conjugated with trastuzumab against human epidermal growth factor receptor 2 (HER2) as a target for breast cancer therapy exhibited an increase in the effectiveness of therapy as seen from the increase in the resulting docking score. The potential for increasing the therapeutic effectiveness of this combined particle preparation was predicted to be obtained in combined particles with a small size, with the consequence of decreasing the spontaneity of the reaction to HER2.

## Figures and Tables

**Table 1:** Composition of packaging particle structure of the combination of α-mangostin and chitosan.

Structure Radius	Number of Molecules			
	α-Mangostin	Chitosan	Sodium Ions	Tripolyphosphate Ions
50 Å	60	161	469	94
75 Å	203	545	1.582	316
100 Å	480	1.291	3.749	750

**Table 2:** Structural preparation results of α-mangostin, chitosan, and sodium tripolyphosphate.

Compound	Chemical Formula	3D Structure
α-mangostin	C_24_H_26_O_6_	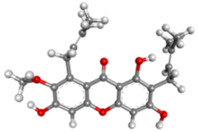
Chitosan-ionated	C_56_H_112_N_9_O_39_^9+^	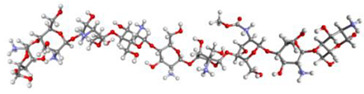
Sodium ion	Na^+^	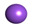
Tripolyphosphate ion	P_3_O_10_^5−^	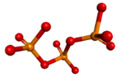

**Table 3:** Evaluation of trastuzumab modeling results.

Model	%Similarity	GMQE	QMEAN |Z-Score|
Trastuzumab	82.60%	0.85	0.05

**Table 4:** Hydrogen bond interactions of amino acid residues in trastuzumab with combined particles of α-mangostin and chitosan at the conjugation stage.

Compound	Docking Score	Hydrogen Bond Interactions
Combined particle (50 Å)	26,606	B:Glu275, B:Trp280, B:Lys291, B:Lys293, B:Tyr303, B:Val306, C:Gln147, C:Asn152, C:Thr197, C:Ser202, C:Ser203, D:Arg295, D:Tyr303
Combined particle (75 Å)	30,532	B:Leu254, B:Ser257, B:His288, B:Ala290, B:Thr292, B:Pro294, B:Glu297, B:Gln298, B:Tyr303, B:Arg304, C:Asp151, C:His198, C:Leu201, C:Ser203, C:Pro204, D:Glu261, D:Thr259, D:Glu296, D:Thr310, D:Glu383, D:Ser386, D:Asn387, D:Gln389, D:Arg419
Combined particle (100 Å)	30,994	C:Ser12, C:Thr109, C:Val110, C:Tyr140, C:Lys149, C:Glu195, C:Gln199, C:Ser202, C:Ser203, C:Thr206, D:Thr259, D:His288, D:Glu296, D:Thr310, D:Gln389, D:Pro390, D:Tyr439, D:Gln441

**Table 5:** Molecular docking simulation results of the preparation and its standards against HER2.

Compound	Docking Score	Area	ACE (kcal/mol)
α-mangostin	5416	683.30	−420.39
Chitosan	10,378	1504.70	−643.54
Tripolyphosphate ion	2688	309.20	71.12
Trastuzumab	21,440	3853.40	196.60
Combined particle (50 Å)	23,044	3663.40	−875.02
Combined particle (50 Å) conjugated with trastuzumab	26,872	4146.30	−582.33
Combined particle (75 Å)	27,858	4008.60	−766.09
Combined particle (75 Å) conjugated with trastuzumab	26,520	4044.80	−580.04
Combined particle (100 Å)	29,884	4389.40	−279.12
Combined particle (100 Å) conjugated with trastuzumab	29,996	4643.70	−424.13

**Table 6:** Molecular docking simulation results of the preparation and its standards against HER2.

Compound	Hydrogen Bond Interactions
α-mangostin	-
Chitosan	Val3, Thr5, Tyr281, Tyr387, Leu414, Ser441
Tripolyphosphate ion	Thr5
Trastuzumab	Asn89, Glu216, Tyr252, Cys289, Glu330
Combined particle (50 Å)	Thr83, Asp88, Asn89, Asn155, Leu157, Gln217, Val250, Tyr252, Thr256, Asp285, His296, Asn297, Lys311, Cys312, Ser313, Lys314, Arg318, Glu326, His327, Glu330
Combined particle (50 Å) conjugated with trastuzumab	Gln2, Leu249, Tyr267, Thr275, Ala276, Thr306, Asp461, Arg465, Arg495, Val507, Cys509, Gln511, Val533, Asn534, Arg536, Gly550, Phe555, Asp560, Glu597
Combined particle (75 Å)	Lys153, Gln156, Leu157, Ala158, Cys202, Gln217, Leu244, Tyr252, Asp255, Thr256, Val292, Cys293, Cys316, Ala317, Val319, Tyr321, His327
Combined particle (75 Å) conjugated with trastuzumab	Thr223, Ser239, Leu249, Met260, Pro263, Arg266, Ser272, Thr275, Tyr279, Asn508, Cys509, Ser510, Gln511, Asn534, Ala535
Combined particle (100 Å)	Thr1, Asp22, Gln29, Gln32, Asn46, Ser50, Gln53, Arg76, Cys190, Thr223, Asp229, Thr275, Asp461, Gln462, Phe464, Leu471, His473, Gln491
Combined particle (100 Å) conjugated with trastuzumab	Thr1, Gln29, Gln32, Asn46, Ser50, Gln53, Arg76, Glu188, Cys190, Ser192, Thr223, Asp229, Cys230, Asn237, Thr275, Asp461, Phe464, Leu471, His473, Glu479, Gln491, Arg495, Ser510

## Data Availability

The data presented in this study are available on request from the corresponding authors.
